# A Health Threat from Farm to Fork: Shiga Toxin-Producing *Escherichia coli* Co-Harboring *bla*_NDM-1_ and *mcr-1* in Various Sources of the Food Supply Chain

**DOI:** 10.3390/pathogens13080659

**Published:** 2024-08-06

**Authors:** Ayesha Sarwar, Bilal Aslam, Muhammad Hidayat Rasool, Mounir M. Salem Bekhit, James Sasanya

**Affiliations:** 1 Institute of Microbiology, Government College University Faisalabad, Faisalabad 38000, Pakistan; ayesha.sarwar@gcuf.edu.pk (A.S.); drmhrasool@gcuf.edu.pk (M.H.R.); 2 Department of Pharmaceutics, College of Pharmacy, King Saud University, P.O. Box 2457, Riyadh 11451, Saudi Arabia; mbekhet@ksu.edu.sa; 3 International Atomic Energy Agency, P.O. Box 100, 1400 Vienna, Austria; j.sasanya@iaea.org

**Keywords:** AMR, food supply chain, non-O157, MDR, STEC

## Abstract

The dissemination of resistant pathogens through food supply chains poses a significant public health risk, spanning from farm to fork. This study analyzed the distribution of Shiga toxin-producing *Escherichia coli* (STEC) across various sources within the animal-based food supply chain. A total of 500 samples were collected from livestock, poultry, the environment, fisheries, and dairy. Standard microbiological procedures were employed to isolate and identify *E. coli* isolates, which were further confirmed using MALDI-TOF and virulence-associated genes (VAGs) such as *stx1, stx2, ompT, hylF, iutA, fimH, and iss*. The phenotypic resistance patterns of the isolates were determined using the disc diffusion method, followed by molecular identification of antibiotic resistance genes (ARGs) through PCR. STEC were subjected to PCR-based O typing using specific primers for different O types. Overall, 154 (30.5%) samples were confirmed as *E. coli*, of which 77 (50%) were multidrug-resistant (MDR) *E. coli*. Among these, 52 (67.53%) isolates exhibited an array of VAGs, and 21 (40.38%) were confirmed as STEC based on the presence of *stx1* and *stx2*. Additionally, 12 out of 52 (23.07%) isolates were identified as non-O157 STEC co-harbouring *mcr-1* and *bla*_NDM-1_. O26 STEC was found to be the most prevalent among the non-O157 types. The results suggest that the detection of STEC in food supply chains may lead to serious health consequences, particularly in developing countries with limited healthcare resources.

## 1. Introduction

The misuse of antimicrobials in food-producing animals (FPAs), either for growth promotion or as a preventive measure, has created selection pressure that fosters the development of resistant bacterial strains. These resistant bacteria can then spread to humans through the food supply chain, either by consuming contaminated food, direct contact with FPAs, or indirectly through animal waste that contaminates production systems, agriculture, aquaculture, and the environment [1]. The complexity of animal-based food chains, due to the extensive diversity in production, processing, packaging, and transportation, often leads to the persistence and recurrence of microbes responsible for foodborne illnesses [2].

Foodborne infections (FBIs) are serious global health concerns, resulting in substantial socioeconomic impact and contributing to significant morbidity and high mortality rates across various age groups worldwide. FBIs are predominantly caused by specific microbial pathogens or other food contaminants such as bacteria, fungi, and mycotoxins. Additionally, environmental contaminants can contribute to these infections by contaminating food during production and transportation [3]. Among the most prevalent pathogens causing FBIs globally are Shiga toxin-producing *E*. *coli* (STEC), along with *Salmonella, Campylobacter, and Listeria* [4]. STEC, a zoonotic pathotype of *E. coli*, is particularly noteworthy for its role in causing diarrhea, hemorrhagic colitis, and hemolytic uremic syndrome.

However, the emergence of non-O157 STEC in recent years has been sporadically reported and is considered a potential health threat [5]. Various serogroups of non-O157 STEC, including O26, O103, and O145, have been prevalent in different outbreaks of STEC. Stx produced by STEC consists of two distinct immunodominant epitopes: Stx1 and Stx2. These Stx toxins play a crucial role in the virulence of STEC within the host, contributing to the onset of infection. Shiga toxins act as ribotoxins, inhibiting protein synthesis within eukaryotic host cells and inducing apoptosis [6]. Identifying specific research gaps in the context of MDR STEC transmission within food supply chains is critical for developing targeted interventions to mitigate the risk of foodborne infections [7]. In recent years, various studies have highlighted the public health significance of non-O157 STEC occurrences worldwide [8,9]. Food-producing animals (FPAs) are recognized reservoirs of STECs, and the characterization of STEC strains from a variety of domesticated animals has been documented [9]. Previously, MDR *E. coli* has been reported in poultry, fresh, and frozen meat [10,11]. Building on this foundation, the present study aims to investigate MDR STEC across various sources within the food supply chain. However, there is limited data available on STEC transmission from farm to fork in Pakistan, likely due to inadequate surveillance and monitoring programs.

## 2. Materials and Methods

### 2.1. Ethical Approval

The present study was conducted following approval from the Institutional Review Board (IRB) and Ethical Review Committee (ERC) (Ref No. GCUF/ERC/137, dated 3 February 2023) at the Government College of the University Faisalabad. Samples were collected with prior permission and written consent from stakeholders. All experiments were conducted at the One Health AMR laboratory (OH-AMR lab) at the Institute of Microbiology, Government College, University Faisalabad. The Matrix-assisted Laser Desorption/Ionization-Time of Flight (MALDI-TOF) experiment was carried out at the National Institutes of Health, M4QP + GW7 Islamabad, Pakistan.

### 2.2. Collection, Processing, and Transportation of Sample

Food samples were collected and divided into five categories livestock products, poultry, environmental samples, fisheries, and dairy which were further divided into sub-categories as mentioned Overall, a total of 500 samples were collected in sterile containers from various food and animal-related sources. These included livestock samples (n = 75), such as chicken, meat, and veal; poultry samples (n = 75), including chicken, cloacal/anal swabs, and droppings; environmental samples (n = 125) from slaughterhouses, open markets, dairy and poultry waste, and various transport vehicles; fisheries samples (n = 100) comprising fish, shrimps, market waste, and transport vehicles; and dairy samples (n = 125), including raw milk, yogurt, dairy cream, cheese, and outlet waste. Samples were preserved in peptone water and transported in ice bags to the Institute of Microbiology’s laboratory for further investigation (Table 1).

### 2.3. Isolation and Purification of E. coli

Enriched samples were streaked on MacConkey agar and Eosine Methylene Blue (EMB) agar (OXOID^®^, Basingstoke, UK), and incubation was done for 24 h at 37 °C. Samples were processed for cultural and morphological characteristics. Furthermore, isolates were subjected to Biochemical identification using an API 20E kit (Biomeurex, Craponne, France) according to the manufacturer protocol.

### 2.4. MALDI-TOF

All the isolates were confirmed through MALDI-TOF-based VITEK^®^ MS V3.2 (Biomeurex, Craponne, France). All the procedure was carried out as per guidelines and recommendations available in the manufacturer manual and protocol. The cyanohyrodxycinamic acid (CHCA @ 0.01 µL) was used as a matrix for the isolates. Whereas ATCC™ *E. coli* (8739) was kept in control. The slides for VITEK^®^ MS were prepared by using Vitek^®^ PICKME NIBS (Biomeurex, Craponne, France), ATCC™ control, and the isolates were inoculated and directed circles, correspondingly. Subsequently, CHCA (0.01 µL). was added to make the tested sample ready to be interpreted through MYLA^®^ software v. 4.9.1 (Biomeurex, Craponne, France).

### 2.5. Detection of VAGs

Detection of various virulence-associated genes (VAGs) in *E. coli* (*stx1*, *stx2*, *ompT*, *hylF*, *papC*, *eae*, *ampC, traT*, *fimH*, and *iss*) was performed using PCR with specific primers [12,13,14,15,16,17,18,19] (see Supplementary Materials). DNA extraction was carried out using the GeneJET Genomic DNA purification kit K0722 (Thermo Scientific™) following the provided protocol.

PCR for VAGs was conducted on a Thermo-cycler:48 Biomerta™ (Göttingen, Germany) under specific conditions and respective annealing temperatures (Supplementary Materials). The reaction setup included initial denaturation of template DNA at 94 °C for 5 min, followed by 30 cycles of DNA denaturation at 94 °C for 1 min, primer annealing for 55 s, and extension at 72 °C for 60 s. A final extension step at 72 °C for 10 min was performed. The PCR reaction mixture was prepared according to the manufacturer (Ambion-AM9932, Thermo Scientific™, Waltham, MA, USA). PCR amplicons were visualized by running 1.5% agarose gel electrophoresis (CSL-AG500; CLEAVER SCIENTIFIC^®^, Rugby, UK) and observed under a gel transilluminator (BioRad, Hercules, CA, USA).

### 2.6. Antibiotic Susceptibility Testing

Antibiotic resistance profiling of *E. coli* isolates (n = 52) was conducted using the Kirby–Bauer disc diffusion assay following the 2021 CLSI guidelines. The antibiotics tested are included, as mentioned in Table 6. *E. coli* ATCC™ 8739 served as a quality control during the experiment. Minimum inhibitory concentrations (MICs) were determined using the Broth microdilution method (BMD), as per CLSI guidelines, except for colistin and tigecycline, which were assessed following EUCAST-CLSI and FDA recommendations [20].

### 2.7. Phenotypic Confirmation of Colistin Resistance

The Rapid Polymyxin Test (RPT) was carried out for the phenotypic confirmation of colistin-resistant isolates as described previously [21]. Polymyxin stock solution was prepared in Muller Hinton Broth (MHB) with colistin (Oxoid™), as a final concentration of 0.2 mg/mL was reached. Afterward, rapid polymyxin NP solution (RPS) was prepared by adding 6.25 g of MHB and 0.0125 g phenol red, the pH of the RPS was adjusted to 6.7. Moreover, sterilized filtered D + glucose (1%) was also added in RPS. At the start of the experiment, colistin was dispensed in RPS to get a solution having colistin @ 5 μg/150 μL. Bacterial inoculum was prepared from freshly grown *E. coli* culture; bacterial colonies were resuspended to obtain 3.5 McFarland standard for further procedure according to EUCAST recommendation. All the isolates that grew in the presence of colistin were classified as colistin-resistant.

### 2.8. Carbapenemase Nordmann-Poirel CLSI (CarbaNP CLSI) Test

CLSI recommended CarbNP assay was carried out to confirm carbapenem-resistant isolates [22]. Two Eppendorf tubes were prepared and labeled separately by adding 20 mM Tris-HCl buffer (100 µL) in each. Moreover, two solutions were prepared and named Solution A and Solution B. Solution A was prepared with 0.1 mm/L ZnSO_4_ and 0.5% phenol red indicator, and the pH of Solution A was kept at 7.8. To prepare solution B, 6 mg/mL of imipenem was mixed in Solution A. Afterwards, Solution A was dispensed into Tube 1, and Solution B was added in Tube 2. Both tubes were incubated for 2 h at 37 °C. The appearance of a yellow color in Tube 2 was interpreted as positive for the CarbNP test.

### 2.9. Molecular Characterization of ARGs

Along with phenotypic-resistance profiling, various ARGs were identified, including, ESBLs (CTX-M, SHV, TEM, OXA, and CMY), MBLs (NDM, KPC, OXA, VMP, and IMP), and Qnrs (qnrS, qnrB, qnrA, gyrA, gyrB, sul1, sul2, tetA, tetB, mcr-1, and mcr-11). This was performed with the help of PCR using specific primers [23,24,25,26,27,28,29,30,31] (see Supplementary Materials). The DNA was extracted using a genomic DNA purification kit, called K0722 (Thermo-Scientific™, Waltham, MA, USA). A total of 25 µL of the reaction mixture for PCR was used, which comprised 5 µL of DNA template, 10 µL of Green DreamTaq Mix (Thermo Fisher Scientific, Waltham, MA, USA), and F&R primers (100 pM), 1 µL each. A total of 8 µL of SuperQ nuclease-free water was added to achieve the desired volume (25 µL). Lastly, a total of 1.5% agarose (CSL-AG500; CLEAVER SCIENTIFIC^®^, Rugby, UK) gel electrophoresis was used to observe the PCR products’ instructions.

### 2.10. O Typing

Shiga toxins (*stx1, stx2*), which produce *E. coli* (STEC; n = 21), were subjected to O typing through PCR using specific primers for various O types, i.e., O26,103,111,121, and O145 [32] (see Supplementary Materials). Conventional PCR was performed as described previously. A total of 25 µL PCR mix composed of 4–5 µL of template DNA, 8 µL of Green DreamTaq Mix (Thermo Fisher Scientific, USA), 1 µL of each primer (F&R), and 10 µL of SuperQwater was added to reach the required volume. A PCR reaction was conducted with the following conditions: 30 cycles of denaturation at 94 °C for 35 s, annealing at 55 °C for 35 s, and extension at 72 °C for 1 min, followed by final extension for 10 min. Furthermore, agarose (CSL-AG500; CLEAVER SCIENTIFIC^®^, Rugby, UK) gel (1.5 % with 1 mg/mL ethidium bromide) was prepared for electrophoresis to examine the PCR products.

### 2.11. Statistical Analysis

The particulars were combined in Excel (Microsoft Office 365) spreadsheets for various different statistical analyses. The relationship between variables from different sample sources was studied through correlation and linear regression. The strength of the existing relationship among variables was quantified. For comparison of means, data was subjected to analysis of variance (ANOVA), to find out if the associated source means were significantly different, and *p*-value < 0.05 was set as significant.

## 3. Results

### 3.1. Distribution of E. coli from Various Food Sources

A total of 154 out of 500 (30.8%) food samples tested positive for *E. coli*, sample distribution showed that among several groups, poultry and fisheries acquired significantly higher numbers of *E. coli*. The highest prevalence of *E. coli* was observed in poultry 38.67% followed by Fisheries 32%. Additionally, environmental and dairy samples exhibited 29.6%, while 25.37% *E. coli* was detected in livestock samples. Further distribution of the prevalence of samples and *E. coli* per category is given in (Table 2).

### 3.2. Distribution of Non-O157 STEC among Various Sample Sources

Overall, our VAGs confirmed 21/52 (40.38%) isolates were observed as STEC. According to distribution, the highest distribution of STEC was 75%, observed in beef samples followed by mutton, cloacal/anal swabs, chicken meat, veal, and poultry droppings, i.e., 42.85%, 40%, 27.27%, and 25%, respectively. Furthermore, among STEC, a total of 12 (57.14%) were found to be non-O157 STEC co-harboring *bla*_NDM-1_ and *mcr-1*. Likewise, various sample sources exhibited results as in non-O157 STEC co-harboring *bla*_NDM-1_ and *mcr-1*. (Table 3; Supplementary Materials).

A total of 21 (40.38%) STEC samples were observed for their respective O types, among them, 11 (52.38%) isolates belong to O_26m,_ including 4 (36.36%) from a beef sample and 2 (18.18%) from chicken, cloacal/anal swabs, and poultry droppings. While 1 (33.33%) and 2 (50%) non-O157 STEC co-harboring *bla*_NDM-1_ and *mcr-1* isolates were detected with O103, O121, O111, and O145, respectively, in poultry, livestock category and slaughterhouse samples (Table 4; Supplementary Materials).

### 3.3. Distribution of non-O157 STEC Co-Harboring bla_NDM-1_ and mcr-1 among Various Sample Sources

Overall, out of STEC, a total of 12/21 (57.14%) were distinguished as non-O157 STEC co-harboring *bla*_NDM-1_ and *mcr*-1, where sample-wise division was shown the higher number 3 (25%) in chicken meat followed by 2 (16.67%) in beef samples, cloacal/anal swabs and poultry droppings, while 1 (8.33%) was observed in mutton and slaughterhouse samples, details are given in Table 2.

### 3.4. VAGs Detection

**Figure 1:**
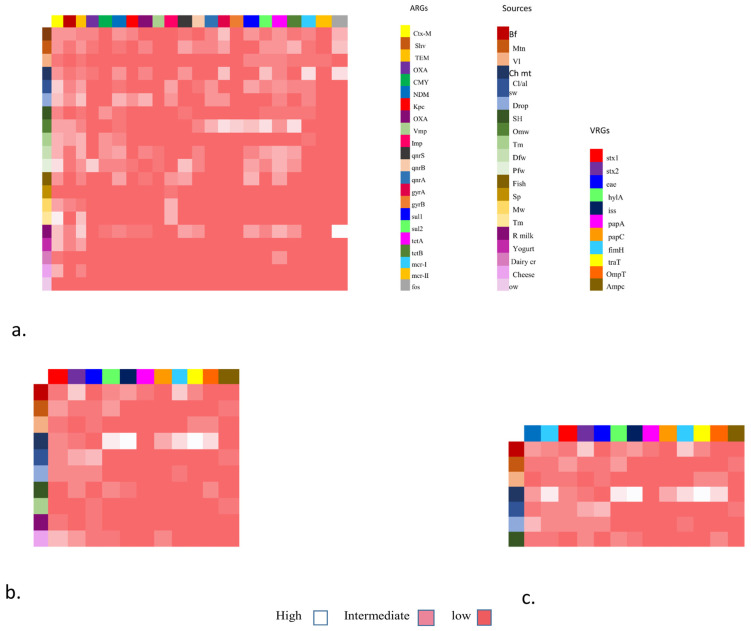
Heat map showing the distribution of antibiotic resistance gene (ARGs), virulence resistance genes (VRGs), and co-existence of NDM-1 and *mcr*-1 associated with VRGs-based *E. coli* detection from various sources.

Overall, 52 (67.53%) isolates have shown the presence of a range of VAGs (Figure 1b). Inclusively, the highest 11 (14.28%) of the chicken isolates carry eight VRGs. Additionally, 8 (10.38%) of beef isolates carry seven ARGs followed by mutton isolates 7 (9.09%) having five VRGs. Moreover, veal and slaughterhouse 4 (5.19%), droppings 8 (10.38%) and cloacal/anal swabs 10 (12.98%) of the isolates carried four VRGs respectively. Furthermore, within all the isolates 23 (29.87%), three marker genes i.e., *ompT, hylF*, and *iss*, were identified. In addition to these, the most common VAGs among *E. coli* were *stx-1* and *stx-2*, followed by *eae*, *papC*, and *traT* (Figure 1).

Furthermore, in addition to the *stx1* and *stx2*, all non-O157 STEC co-harboring *bla*NDM-1 and *mcr*-1 isolates have shown the presence of a range of VAGs including *hylA, eae, iss, papC, papA, papG, tsh, ibeA, iuOD, fimH, traT, ompT, ampC*, etc. (Table 5; Figure 1c). (The abbreviations used for food sources are Bf (beef), Mn (mutton), VL (veal), CH mt (chicken), cl/al sb (cloacal/ansl swab), Drop (droppings), SH (slaughterhouse), OMW (open market waste), Tm (transport means), DFW (dairy farm waste), Pfw (poultry farm waste), fish (fish), SP (shrimp), MW (market waste), Tm (transport mean), R milk (raw milk), Yogurt, Dairy cr (dairy cream), cheese, OW (outlet waste))

The heat map shows the distribution of five major categories of food specimens from various food origins. The *y*-axis shows these origins with colored boxes. Antibiotic resistance genes including *ESBLs*, *MBLs*, *qnrs*, *sul*, *tet*, *mcr (1,11*), and fos are shown in colored boxes on the *x*-axis. These genes were grouped as low, intermediate, and high frequency.

The heat map also shows the placement of virulence genes associated with Fimbrae, mobility, toxin, iron uptake, etc. The *x*-axis shows *E. coli* isolates from various food groups (*y*-axis) mainly livestock (mutton, beef, veal), poultry (chicken, cloacal/nasal swabs and dropping), environment (slaughterhouse and transport means), and dairy (raw milk and cheese) and each is shown as low, intermediate and high on the right side.

The heat map also shows the co-existence of NDM-1 and *mcr*-1 with VRGs-based *E. coli* detection. In this case, VRGs were detected mainly from livestock (mutton, beef, veal), poultry (chicken meat, cloacal/nasal swabs, and droppings), and one environmental sample (slaughterhouse). They are grouped as high, low, and intermediate values and also show the co-occurrence of ARGs (NDM-1 and *mcr-1*).

### 3.5. Resistance Profiling of the Isolates

**Figure 2:**
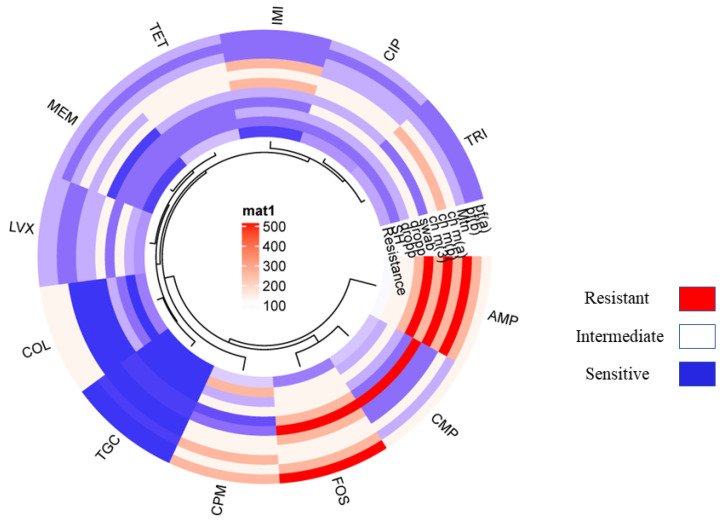
Showing the circular cluster heat map illustrated the Resistance pattern of various standard antibiotics against VRGs based confirmed *E. coli* isolates co-harboring NDM-1 and *mcr-1*. The resistance pattern among *E. coli* isolates harboring NDM-1 and *mcr-1* represented by color and cluster showing overall resistance percentages against antibiotics (AMP, CMP, COL, LVX, MEM, IMI, CIP, TRI, TOC, and FOS).

The resistance pattern of the non-O157 STEC co-harboring *bla*_NDM-1_ and *mcr*-1 isolates showed 100% resistance to ampicillin followed by cefepime. Additionally, chloramphenicol showed 75% tetracycline, 72% trimethoprim, 65% ciprofloxacin, 51% levofloxacin, 45% colistin, and 40% fosfomycin, 10% imipenem and meropenem. The least resistance is shown in the case of tigecycline, which was just 4% (Figure 2; Table 6). Moreover, all non-O157 STEC co-harboring *bla*_NDM-1_ and *mcr-1* have shown positive for CarbaNP and rapid polymyxin confirmation (see Supplementary Materials).

### 3.6. ARGs

**Figure 3:**
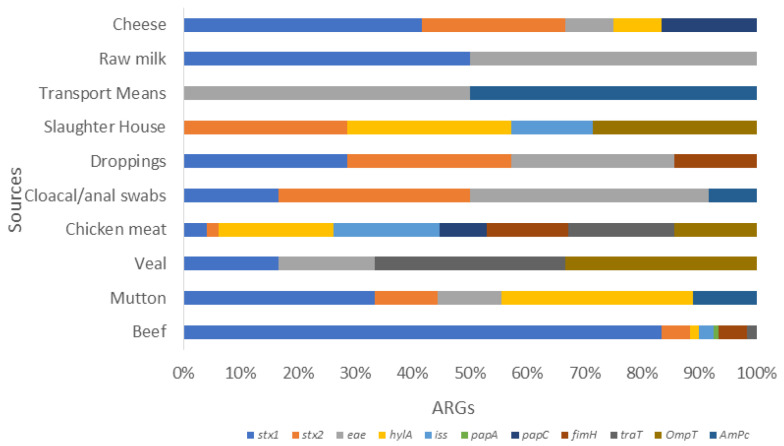
Stacked bar showcasing the prevalence of various VRGs in *E. coli* isolates from various food sources. This stalked bar describes the detection of various VRGs (*stx1*, *stx2*, *eae*, *hylA*, *iss*, *pap A*. *papC*, *papG*, *fimH*, *traT*, *ompT*, and *ampC*) shown in various colors set against selected food sources from livestock. It shows poultry, dairy, and environmental sample categories. The highest prevalence rates were *stx1* in cheese, followed by *stx2* in slaughterhouse samples. The chicken meat carried more *hylA*, *iss*, *traT*, *ompT*, and *papC*, whereas *papA* and *fimH* were more prevalent in beef. This was followed by *ampC*, which was high in various transport means from environment samples.

In general, a total of 77/154 (50%) isolates showed the presence of different ARGs (Figure 1a). Among various ESBLs, the following detection patterns were observed: *CTX-M* (52.94%), *SHV* (66.67%), *TEM* (83.33%), *OXA* (77.78%), and *CMY* (75%). Regarding detection rate of MBLs, they showed *bla*_NDM_ (62.5%) followed by *bla*_IMP_ (71.42%), *bla*_OXA_ (62.50%), and *bla*_KPC_ (25%). The rate was the lowest for *bla*_VMP_ (11.11%). Regarding *Qnr* genes, *qnrS* was observed in 66.67% of isolates, followed by *qnrA* (50%) and *qnrB* (28.57%) (Figure 3). Likewise, the detection rate of *gyrA, gyrB, sul1, and sul2* was 80%, 70%, 80%, and 50% respectively. Furthermore, the rate of *tetB* and *tetA* was 80% and 66.67%, respectively.

(a) The scatter plot shows the presence of ESBL genes in various food sources. A high number of *bla*CTX-M was detected in fisheries via transport means whereas *bla*SHV is more in beef. Among *bla*TEM and *bla*OXA, *bla*CMY showed the highest prevalence in raw milk, poultry waste, and slaughterhouse samples, respectively.
(b) The scatter plot in MBLs showcases the highest prevalence of *bla*NDM-1 and *bla*OXA in poultry droppings and the lowest in poultry farm waste while *bla*IMP in fisheries market waste.
(c) The scatter plot shows *qnrS*. *qnrS* was highest in poultry farm waste, followed by *qnrA* which was found to more prevalent in open market waste, along with *qnrB*, which was found to be more prevalent in raw milk. Out of the different types of *gyrs*, *gyrA* is more prevalent than *gyrB* and was detected in higher quantities in open market waste.
(d) The scatter plot also elaborates on the prevalence of *sul* and *tet* genes. Here, *sul1* and *sul2* are shown to be more prevalent in environmental waste samples. Meanwhile, *tetA* is higher in dairy waste. Consider this in comparison to *tetB*, which is found more in open market waste.

## 4. Discussion

*E. coli* emerges as a key protagonist due to its ubiquity in various ecosystems and its ability to readily exchange genetic material containing resistance genes. This characteristic illustrates how antimicrobial use and resistance development in one sector can swiftly disseminate and manifest in other domains [33]. Therefore, studies are needed to elucidate the pathogenicity and resistance paradigm of *E. coli* originating from food sources, which may play a significant role in mitigating the health impacts caused by such pathogens.

**Figure 4:**
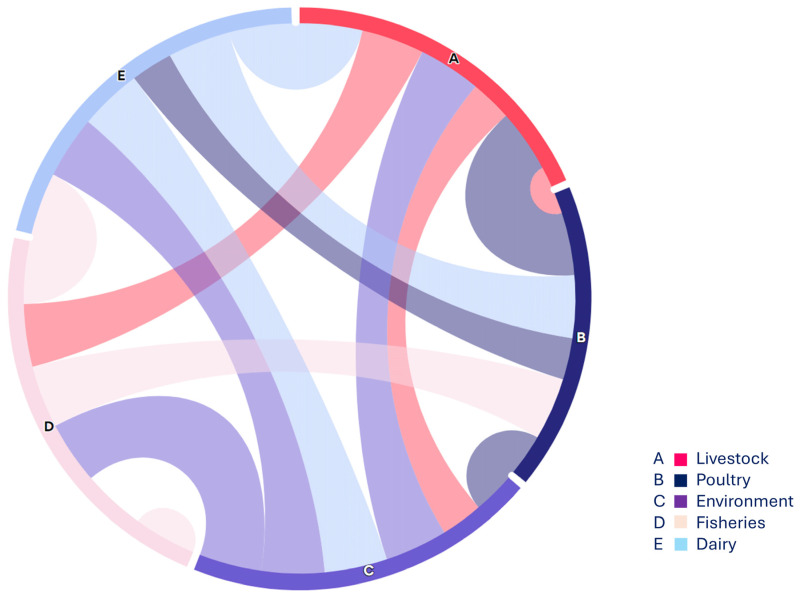
The chord diagram (https://www.everviz.com/, accessed on 18 March 2024) represented the flow between various studied sample sources i.e., nodes. Each source is displayed with an alphabet on the outer circle and arcs between different sources showing the connection and relevance with adjacent sources.

In this regard, the present study focused on non-O157 STEC strains co-harboring blaNDM-1 and mcr-1 across various sources, highlighting the complexity of the food supply chain (Figure 4). Overall, 2.4% (12/500) of non-O157 STEC strains co-harboring mcr-1 and blaNDM-1 were detected in all studied sources, indicating a concerning distribution of non-O157 STEC within the food supply chain. While STEC O157 strains are often associated with outbreaks, recent reports suggest that non-O157 STEC strains also possess the ability to cause illnesses such as HUS [34]. Consequently, research on the distribution, incidence, and pathogenicity of non-O157 STEC has gained significant attention due to recent outbreaks involving these serogroups. A recent study reported on the antimicrobial resistance (AMR) profiles of non-O157 STEC from both human and domesticated animal sources, revealing a notable incidence of non-O157 STEC strains co-harboring the mcr-1 gene [5], which supports the findings of the current investigation. Similarly, our findings align with previous work [31] that identified STEC strains in poultry in Nigeria, and with another study [35], which reported the detection of STEC (stx1, stx2) in poultry droppings and cloacal swabs. These results are consistent with findings reported from various low- and middle-income countries (LMICs) as well as China [36,37].

Overall, 21 out of 500 isolates (4.2%) were confirmed as STEC, with both stx1 and stx2 detected in these isolates. Previously reported data on non-O157 STEC have shown incidence rates ranging from 5% to 10% [38,39]. However, due to geographical variations, differences in food categories, processing methods, transportation practices, and STEC identification protocols, direct comparisons of these findings are challenging. Nevertheless, the current results corroborate previous data and offer insights into the relative distribution of STEC among the studied sources. Moreover, available literature indicates that among various non-O157 STEC serogroups, O26 is the most prevalent. Consistent with this, the current study found that O26 was the most common serogroup, comprising 52% of the isolates among the studied samples. Recently, a study in China reported the presence of O26 in non-O157 STEC isolated from retail foods [40], further supporting our findings. Similarly, the detection of non-O157 STEC in this investigation aligns with previous reports from different regions, underscoring the increasing concern over foodborne illnesses associated with non-O157 STEC [41].

All STEC isolates exhibited an array of virulence-associated genes (VAGs), including stx1 and stx2. These findings are consistent with previous reports documenting the distribution of stx1 and stx2 among STEC isolated from diverse food sources. Epidemiological and clinical investigations have indicated that the presence of stx2 is crucial for causing hemolytic uremic syndrome (HUS), and strains carrying additional VAGs can lead to severe illnesses [42]. Furthermore, the findings of the present study align with published research indicating that non-O157 STEC originating from food sources exhibit a variety of VAGs [42]. Recent studies from Pakistan have highlighted that the most common VAG among STEC isolated from food animals is the stx2 gene (24.81%) [43]. Similarly, a study conducted in the USA reported a prevalence of stx genes (16.6%) in STEC isolated from food animals [44]. These findings are consistent with studies from several European countries, such as Hungary, Poland, Switzerland, and Austria, which have also reported similar patterns of VAGs in various meat samples [45].

The isolates in this study exhibited considerable resistance to the tested antibiotics. The statistically significant resistance pattern (Supplementary Materials) observed aligns with previously reported findings concerning STEC from food samples [46]. Antibiotic resistance, particularly against quinolones, may enhance the virulence of STEC by facilitating the induction of stx1 or stx2 genes. The significant resistance observed among the isolates underscores the concern that unregulated and inappropriate antimicrobial use contributes to the emergence and spread of antimicrobial resistance (AMR) and resistant bacterial strains.

**Figure 5:**
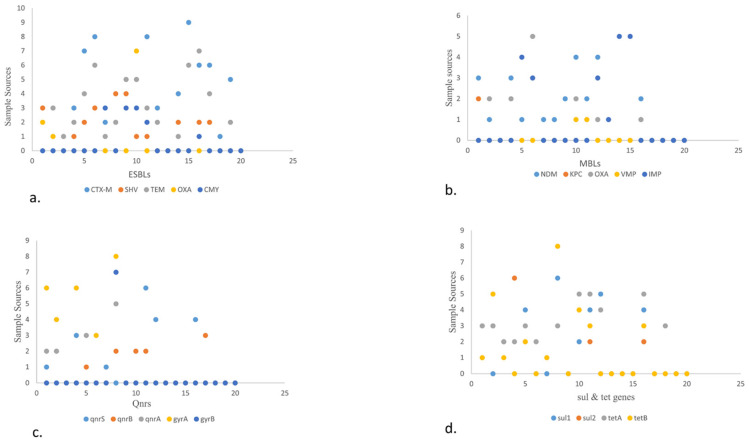
Scatter plot describing the sample sources against different VRGs from different food origins. Scatter plot showing the sample sources and ARGs in the form of numerical value. The *Y*-axis shows the sample source. 0–9 depicts beef, mutton, veal, chicken, cloacal/anal swabs, droppings, environmental samples (slaughterhouse, open market, and transport waste, dairy and poultry waste), fish, shrimps, market waste, transport means, and dairy (raw milk, yogurt, dairy cream, cheese, and outlet waste) and *x*-axis showing ARGs (*ESBLs*, *MBLs*, *qnrs*, *tet*, and *sul*).

A range of antibiotic resistance genes (ARGs) were detected in the studied isolates, consistent with findings from previous studies [17,47,48,49,50,51] (Figure 5). Additionally, metallo-beta-lactamases (MBLs) were prevalent, detected in up to 68 out of 500 isolates (13.8%), which correlates with findings reported in clinical isolates [52]. Another study from Nigeria highlighted the presence of *bla*_VIM_ specifically in *E. coli* isolated from diarrheal samples associated with foodborne illnesses [53].

The current research indicates a significant correlation between the antibiotic resistance profiles of STEC isolates and the presence of ARGs (Supplementary Materials). For instance, the highest resistance among *E. coli* isolates was observed against ampicillin, with blaTEM detected in 83% of the isolates. Similarly, 40% of the isolates exhibited resistance to colistin, with *bla*_IMP_ detected in 40% of these resistant strains [54]. These findings align with previous work demonstrating high resistance patterns and the detection of various ARGs among STEC isolated from food-producing animals.

In the poultry and livestock sectors, common practices such as indiscriminate antibiotic use as growth promoters and within food production chains significantly contribute to the dissemination of ARGs, contaminating the environment and posing serious health hazards to ecosystems [55]. This practice facilitates the emergence of antibiotic-resistant strains, including those harboring mcr-1 and *bla*_NDM-1_ genes. The current study supports this notion, with nearly 40% of STEC isolates exhibiting resistance to colistin, a last-resort antibiotic for Gram-negative bacteria (GNB). Similar observations have been reported globally: in Malaysia, a 100% detection rate of mcr-1 among *E. coli* isolated from poultry and poultry meat showed resistance to colistin [56]. In Bangladesh, a study reported 55.77% of mcr-1-harboring *E. coli* in commercial poultry meat [57]. Studies from the Czech Republic and China also reported significant levels of colistin resistance among *E. coli* isolates from meat samples and river water, respectively [58,59].

In developing countries, high levels of colistin and blaNDM-1 resistance, particularly in the poultry and livestock sectors where the environment serves as a major reservoir, underscore the urgent need for action. There should be a complete ban on the indiscriminate use of antibiotics as growth promoters in poultry and livestock production to mitigate the emergence and spread of various antibiotic-resistant strains.

AMR monitoring and surveillance of foodborne pathogens within the food supply chain represent a multifaceted and complex challenge. However, the present study has several limitations that should be acknowledged. Firstly, the geographical distribution of the samples was limited, which may restrict the generalizability of the findings. Future research should adopt a more comprehensive approach by including samples from various geographical locations with diverse socioeconomic conditions and food production practices. Moreover, the current study lacks detailed genomic analysis of STEC isolates, such as whole genome sequencing, sequence typing, and functional genomics. Future investigations should incorporate these advanced genomic techniques to enhance our understanding of STEC dynamics, particularly in relation to antimicrobial resistance.

## 5. Conclusions

In conclusion, this study provides valuable insights into the molecular characteristics of non-O157 STEC isolated from diverse food sources. Our findings highlight the impact of indiscriminate antibiotic use in poultry and livestock farms, not only in disrupting the food supply chain but also in facilitating the transmission of resistant superbugs, such as STEC carrying mcr-1 and *bla*_NDM-1_, to the wider community through contaminated environments. The pervasive presence of these resistant *E. coli* strains throughout the food supply chain is concerning and demands immediate action. It is imperative to implement measures to ensure the safety and quality of food from farm to fork. Addressing these challenges requires coordinated efforts across sectors to promote judicious antibiotic use, enhance surveillance of antimicrobial resistance, and implement stringent food safety protocols. By taking proactive steps now, we can mitigate the risks posed by antimicrobial resistance and safeguard public health.

## Figures and Tables

**Table 1:** The collection and distribution of samples from various food origins.

Sr. No.	Specimen Category	Sources of Samples	Number of Collected Samples	Total Samples
1	Livestock products	Beef	25	75
		Mutton	25	
		Veal	25	
2	Poultry	Chicken	25	75
		Cloacal/anal swabs	25	
		Droppings	25	
3	Environmental samples	Slaughterhouse	25	125
		Open market waste	25	
		Transport Means	25	
		Dairy farm waste	25	
		Poultry farm waste	25	
4	Fisheries	Fish	25	100
		Shrimps	25	
		Market waste	25	
		Transport means	25	
5	Dairy	Raw milk	25	125
		Yogurt	25	
		Dairy cream	25	
		Cheese	25	
		Outlet waste	25	
	Grand Total			500

**Table 2:** Prevalence and Distribution of *E. coli* from selected food categories.

Specimen Category	Sources	Collected Samples	Positive Sample	Distribution of *E. coli* Positive Samples	Distribution of *E. coli* from Selected Categories	*p*-Value
Livestock products	Beef	25	8	32%	19/75 (25.34%)	0.001 *
	Mutton	25	7	28%		
	Veal	25	4	16%		
Poultry	Chicken meat	25	11	44%	29/75 (38.67%)	
	Cloacal/anal swabs	25	10	40%		
	Droppings	25	8	32%		
Environmental samples	Slaughterhouse	25	4	16%	37/125 (29.6%)	
	Open market waste	25	10	40%		
	Transport means	25	6	24%		
	Dairy farm waste	25	8	32%		
	Poultry farm waste	25	9	36%		
Fisheries	Fish	25	6	24%	32/100 (32%)	
	Shrimps	25	2	8%		
	Market waste	25	7	28%		
	Transport means	25	17	68%		
Dairy	Raw milk	25	12	48%	37/125 (29.6%)	
	Yogurt	25	9	36%		
	Dairy cream	25	4	16%		
	Cheese	25	7	28%		
	Outlet waste	25	5	20%		
Grand Total		500	154		154/500 (30.8%)	

* Highly significant results among isolates from various sample sources. Summary: Multiple R = 0.87; R Square = 0.76; Adjusted R Square = 0.68; SE = 4.15.

**Table 3:** Non-O157 STEC co-harboring *bla*_NDM-1_ and *mcr-1* among various sample sources along with detected O types.

Specimen Category	VAGs Based Confirmed *E. coli*	STEC % out of *E. coli*	Non-O17STEC Co-Harboring *bla*_NDM-1_ & *mcr-1*	O Type	Statistical Analysis
Beef	8(32%)	6/8(75%)	2/6(33.33%)	O_26_, O_103_ & O_121_	<0.05 *
Mutton	7(28%)	3/7(42.85%)	1/3(33.33%)	O_26_, O_103_ & O_111_	
Veal	4(16%)	1/4(25%)	1/1(100%)	O_121_	
Chicken meat	11(44%)	3/11(27.27%)	3/3(100%)	O_26_ & O_145_	
Cloacal/anal swabs	10(40%)	4/10(40%)	2/4(50%)	O_26_, O_111_ & O_145_	
Droppings	8(32%)	2/8(25%)	2/2(100%)	O_26_	
Slaughterhouse	4(16%)	2/4(50%)	1/1(100%)	O_103_& O_12_	
Overall	52(29.71%)	21/52(40.38%)	12/21(57.14%)		

* Statistical significance among non-O157 O types.

**Table 4:** Non-o157 O types among STEC isolated from various sources along with co-existence of ARGs.

Sources	STEC	Non-O157 O Types					Co-Existence of ARGs	*p* Value
		O26	O103	O111	O121	O145		
Beef	6	4	1	--	1	--	*bla*_NDM,_*mcr-1*, *bla*_TEM_*, bla*_OXA_*, bla*_KPC_*, bla*_qnrA,_	<0.05 *
Mutton	3	1	1	1		--	*bla*_NDM,_*mcr-1*, *bla*_SHV_*, bla*_OXA_,	
Veal	1	--	--	--	1	--	*bla*_NDM,_*mcr-1*, *bla*_CTX-M_*, bla*_TEM_*, bla*_tetB_	
Chicken meat	3	2	--	--	--	1	*bla*_NDM,_*mcr-1*, *bla*_CTX-M,_*bla*_qnrS,_	
Cloacal/anal swabs	4	2	--	1	--	1	*bla*_NDM,_ *mcr-1, bla*_qnrB,_	
Droppings	2	2	--	--	--	--	*bla*_NDM,_ *mcr-1, bla*_tetA_	
Slaughterhouse	2	--	1	--	1	--	*bla*_NDM,_ *mcr-1, bla*_TEM,_ *bla*_qnrS,_ *bla*_tetA,_ *bla*_Sul2,_ *bla*_tetB_	
Total	21	11	3	2	3	2		

* Results are statistically significant in reference to STEC from beef and poultry droppings with O26.

**Table 5:** Co-existence of *bla*_NDM-1_ and *mcr-1* in VAGs based confirmed *E. coli*.

Sample Source	Positive VAGs in *E. coli*	*NDM*	*mcr-1*	*Stx*					*Pap*						*p* Value
				*stx1*	*stx2*	*Eae*	*hylA*	*Iss*	*papA*	*papC*	*fimH*	*TraT*	*OmpT*	*AmpC*	
Beef	8	3 37.5%	2 25%	1 12.5%	6 75%	ND	2 25%	3 37.50%	1 12.5%	ND	6 75%	2 25%	ND	ND	0.001 *
Mutton	7	1 14.28%	1 14.28%	3 42.85%	1 14.28%	1 14.28%	3 42.85%	ND	ND	ND	ND	ND	ND	1 14.28%	
Veal	4	ND	1 25%	1 25%	ND	1 25%	ND	ND	ND	ND	ND	2 50%	2 50%	ND	
Chicken Meat	11	3 27.27%	8 72.72%	2 18.18%	1 9.09%	ND	8 88.89%	9 81.81%	ND	4 36.36%	7 63.63%	9 81.81%	7 63.63%	ND	
Cloacal /anal Swabs	10	1 10%	2 20%	2 20%	4 40%	5 50%	ND	ND	ND	ND	ND	ND	ND	1 10%	
Droppings	8	5 62.5%	2 25%	2 25%	2 25%	2 25%	ND	ND	ND	ND	1 12.5%	ND	ND	ND	
Slaughter House	4	1 25%	1 25%	ND	2 50%	ND	2 50%	1 25%	ND	ND	ND	ND	2 50%	ND	
Total	52	14	17	11	16	9	15	13	1	4	14	13	11	2	

* Highly significant (*p* value; 0.001), whereas *p* value = < 0.05 from various sample sources ND = not detected.

**Table 6:** Antibiotic susceptibility testing of non-O157, *bla*_NDM-1,_ and *mcr-1*co-harboring STEC isolates.

Antibiotics	Conc.	CLSI EUCAST/FDA Resistance Breakpoint	Beef Samples (n = 2)		Mutton Sample (n = 1)	Veal Sample (n = 1)	Chicken (n = 3)			Poultry Cloacal /Anal Swabs (n = 2)		Poultry Droppings (n = 2)		Slaughterhouse (n = 1)
Ampicillin	10 µg	≥32	128	256	512	256	256	512	256	512	512	256	128	128
Cefepime	30 µg	≥16	256	128	256	256	128	128	32	32	16	128	64	256
Ciprofloxacin	5 µg	≥4	64	32	64	128	64	128	128	64	64	128	64	32
Levofloxacin	5 µg	≥4	64	64	32	32	32	64	64	64	128	32	128	64
Chloramphenicol	30 µg	≥32	128	64	128	64	32	32	512	32	32	64	128	64
Trimethoprim	5 µg	≥16	32	32	64	32	128	256	128	64	32	128	64	32
Imipenem	10 µg	≥4	32	32	32	32	256	128	256	128	64	32	64	32
Meropenem	10 µg	≥4	64	32	64	64	128	64	128	8	8	32	32	32
Colistin	10 µg	≥8	128	128	128	32	4	4	4	4	4	64	32	4
Tetracycline	30 µg	≥16	64	32	64	64	128	128	128	32	64	32	32	32
Tigecycline	15 µg	≥8	4	4	8	8	4	4	8	4	8	4	4	4
Fisfomycin	200 μg	≥64	512	256	128	128	128	256	512	128	256	128	128	128

## Data Availability

The original contributions presented in the study are included in the article/supplementary material, further inquiries can be directed to the corresponding author/s.

## Supplementary Materials

The following supporting information can be downloaded at: https://www.mdpi.com/article/10.3390/pathogens13080659/s1, Table S1: Detail of Primers used in study; Figure S1: Distribution of *E. coli* in various food origin; Figure S2: Linear regression analysis from different food sources.
