# Supplementary material for: A Health Threat from Farm to Fork: Shiga Toxin-Producing Escherichia coli Co-Harboring blaNDM-1 and mcr-1 in Various Sources of the Food Supply Chain
Source: Pathogens. 2024 Aug 6;13(8):659. doi: 10.3390/pathogens13080659 (PMC11357323; doi:10.3390/pathogens13080659)
Supplement: Supplementary file 1 [file pathogens-13-00659-s001.zip › pathogens-3091183-supplementary.pdf]

Supplementary material

Table S1: Detail of Primers used in study

Sr. no	Antibiotics/VAGs/Serogroups	Target	Sequence	Annealing Temp (°C)	Amplicon Size	References
			**ARGs**			
1	β-lactams	*bla*CTX-M-1	F: ATGTGCAGYACCAGTAARGTKATGGC<br>R: TGGGTRAARTARGTSACCAGAAYCAGCGG	61	593	[23-25]
2		*bla*TEM	F: CGCCGCATACACTATTCTCAGAATGA<br>R: ACGCTCACCGGCTCCAGATTTAT	61	445	
3		*bla*SHV	F: CTTTATCGGCCCTCACTCAA<br>R: AGGTGCTCATCATGGGAAAG	61	237	
4		*bla*CMY	F: CTGAC AGCCT CTTTC TCCAC A<br>R: CTACG TAGCT GCCAA ATCCA C	63	1100	
5	Carbapenems	*bla*NDM-1	F: TGCCCAATATTATGCACCCGG<br>R: CGAAACCCGGCATGTCGAGA	59	292	[26-27]
6		*bla*OXA	F: TTGGTGGCATCGATTATCGG<br>R: GAGCACTTCTTTTGTGATGGC	55	743	
7		*bla*KPC	F: TGCAGAGCCCAGTGTCAGTTT<br>R: CGCTCTATCGGCGATACCA	52	880	
8		*bla*IMP	F: GGAATAGAGTGGCTTAATTCTC<br>R: CCAAACCACTACGTTATC	54	624	
9		*bla*VIM	F: GTTTGGTCGCATATCGCAAC<br>R: AATGCGCAGCACCAGGATAG	52	389	
10	Quinolones	*qnrS*	F: GCCAATTGYTACGGKATWGAG<br>R: GACTCTTTCARTGATGCRCC	54	227	[28-29]
11		*qnrB*	F: CGACCTGAGCGGCACTGAAT<br>R: TGAGCAACGATGCCTGGTAG	52	515	[30]
12		*qnrA*	F: TCAGCAAGAGGATTTCTCA<br>R: GGCAGCACTATTACTCCCA	51	627	
13		*gyrA*	F:ATGAGCGACCTTGCGAGAGAAATTACACCG<br>R: TTCCATCAGCCCTTCAATGCTGATGTCTTC	58	659	[31]
14	Sulphonamides	*sul1*	F: TCA CCG AGG ACT CCT TCT TC<br>R:CAG TCC GCC TCA GCA ATA TC	53	435	
15		*sul2*	F: GCGCTCAAGGCAGATGGCAT	53	293	
16	Tetracycline	*tetA*	F: GTAATTCTGAGCACTGTCGC<br>R: CTGCCTGGACAACATTGCTT	55	956	
17		*tetB*	F: CTCAGTATTCCAAGCCTTTG<br>R: ACTCCCCTGAGCTTGAGGGG	55	414	
18	Colistin	*mcr-I*	F: AGTCCGTTTGTTCTTGTGGC<br>R: AGATCCTTGGTCTCGGCTTG	60	320	
			**VAGs**			
1	Shiga toxin	*stx1*	F: CACAATCAGGCGTCGCCAGCGCACTTGCT<br>R: TGTTGCAGGGATCAGTGGTACGGGGATGC	58	606	[15-17]

2		*stx2*	F: CCACATCGGTGTCTGTTATTAACCACACC<br>R:GCAGAACTGCTCTGGATGCATCTCTGGTC	58	372	
3	Enteropathogenic toxin	*Eae*	F: CCCGAATTCGGCACAAGCATAAGC<br>R: CCCGGATCCGTCTCGCCAGTATTCG	57	881	
4	Hemolysin F	*hylF*	F: GGCCACAGTCGTTTAGGGTGCTTACC<br>R: GGCGGTTTAGGCATTCCGATACTCAG	58	450	
5	Increased serum survival	*Iss*	F: CAGCAACCCGAACCACTTGATG<br>R: AGCATTGCCAGAGCGGCAGAA	57	323	
6	Type 1 fimbrin D mannose adhesion	*fimH*	F: TCGAGAACGGATAAGCCGTGG<br>R: GCAGTCACCTGCCCTCCGGTA	58	508	
7	P fimbriae	*papC*	F: TGATATCACGCAGTCAGTAGC<br>R: CCGGCCATATTCACATAA	57	501	
8	F factor transfer gene	*traT*	F: GGTGTGGTGCGATGAGCACAG<br>R:CACGGTTCAGCCATCCCTGAG	64	290	[18-19]
9	outer membrane protein	*Ompt*	F: TCATCCCGGAAGCCTCCCTCACTACTAT<br>R: TAGCGTTTGCTGCACTGGCTTCTGATAC	59	496	
10	ampicillin resistance gene group C	*Ampc*	F: GTGAAGCCRTCTGGTTTGAG<br>R: GCGACATAGCTACCAAATCCG	63	494	
			**O serogroups**			
1	Serogroup O26	ECO26	F: CAATGGGCGGAAATTTTAGA<br>R: ATAATTTTCTCTGCCGTCGC	56	514	
2	Serogroup O103	ECO103	F: TTGGAGCGTTAACTGGACCT<br>R:GCTCCCGAGCACGTATAAAG	56	321	
3	Serogroup O111	ECO111	F: TGTTTCTTCGATGTTGCGAG	56	438	[32]
4	Serogroup O121	ECO121	F: TCCAACAATTGGTCGTGAAA<br>R: AGAAAGTGTGAAATGCCCGT	56	628	

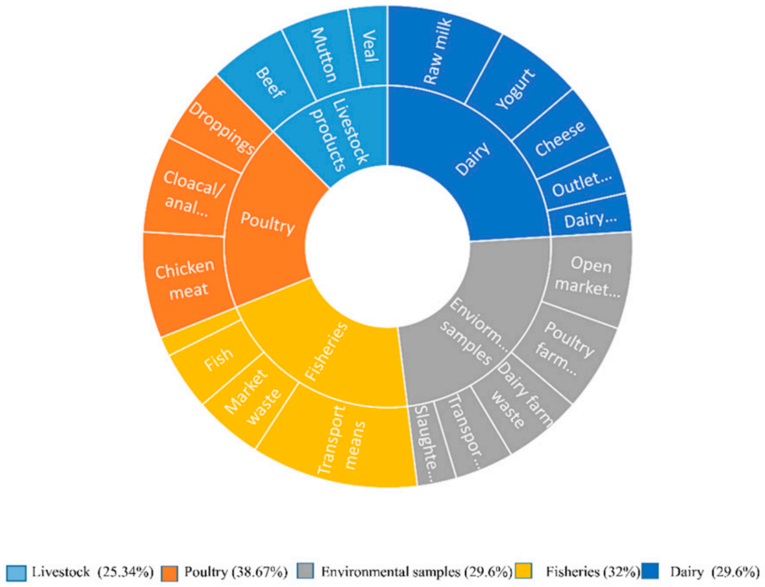

Figure S1: Distribution of *E. coli* in various food origin which depicted by colorful parts of pie chart showed the percentage of *E. coli* in five major categories which then further subdivided according to sample source. The colorful parts of pie chart also showed the percentage from different categories.

Supplementary material: Linear regression analysis from different food sources.

(a) Linear regression/correlation (multiple R = 0.72) showed the co-existence of bla$_{NDM-1}$ and *mcr*-1 in VRGs based confirmed *E. coli*. Here in chicken showing the highest percentage in marker VAGs (*stx1*, *stx2*, *eae*, *hylA*, *fimH*) along with *bla*$_{NDM-1}$ and *mcr*-1 co harboring *E. coli* followed by beef, mutton, poultry droppings and cloacal /anal swabs. As same increase pattern was observed in both cases.

(b) Linear regression of non-O157, *bla*$_{NDM-1}$ and *mcr-1* harboring STEC isolates revealed the correlation among MICs of the isolates from chicken and chicken droppings.

(c) Linear regression shows correlation of different selected sources and various Serotypes. In livestock category (beef samples) exhibited high percentage and same sample sources showing the high level of O26 in STEC cohobring bla$_{NDM-1}$ and *mcr*-1 followed by chicken , cloacal/anal swabs and poultry dropping. Along with other Otypes, O103, O121 were also present along with O111 and O145 among selected food sources showing same rise pattern.

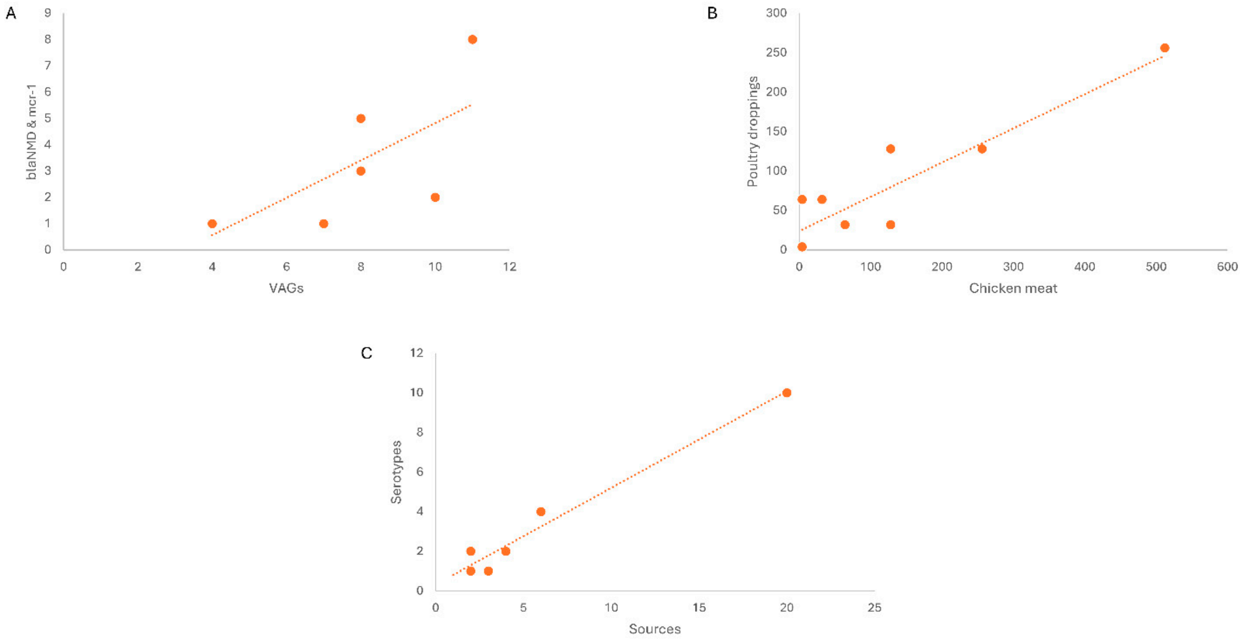

Figure S2: Linear regression analysis from different food sources
